# Model-Based Person Fit Statistics Applied to the Wechsler Adult Intelligence Scale IV

**DOI:** 10.1177/00131644251339444

**Published:** 2025-08-03

**Authors:** Jared M. Block, Steven P. Reise, Keith F. Widaman, Amanda K. Montoya, David W. Loring, Laura Glass Umfleet, Russell M. Bauer, Joseph M. Gullett, Brittany Wolff, Daniel L. Drane, Kristen Enriquez, Robert M. Bilder

**Affiliations:** 1 UCLA, Los Angeles, CA, USA; 2 University of California, Riverside, CA, USA; 3 Emory University, Atlanta, GA, USA; 4 Medical College of Wisconsin, Milwaukee, WI, USA; 5 University of Florida, Gainesville, FL, USA; 6 UCLA David Geffen School of Medicine, and Jane & Terry Semel Institute for Neuroscience and Human Behavior, Los Angeles, CA, USA

**Keywords:** psychometrics, neuropsychology, person fit, PVT

## Abstract

An important task in clinical neuropsychology is to evaluate whether scores obtained on a test battery, such as the Wechsler Adult Intelligence Scale Fourth Edition (WAIS-IV), can be considered “credible” or “valid” for a particular patient. Such evaluations are typically made based on responses to performance validity tests (PVTs). As a complement to PVTs, we propose that WAIS-IV profiles also be evaluated using a residual-based M-distance ( $d_{\mathrm{ri}}^{2}$ ) person fit statistic. Large $d_{\mathrm{ri}}^{2}$ values flag profiles that are inconsistent with the factor analytic model underlying the interpretation of test scores. We first established a well-fitting model with four correlated factors for 10 core WAIS-IV subtests derived from the standardization sample. Based on this model, we then performed a Monte Carlo simulation to evaluate whether a hypothesized sampling distribution for $d_{\mathrm{ri}}^{2}$ was accurate and whether $d_{\mathrm{ri}}^{2}$ was computable, under different degrees of missing subtest scores. We found that when the number of subtests administered was less than 8, $d_{\mathrm{ri}}^{2}$ could not be computed around 25% of the time. When computable, $d_{\mathrm{ri}}^{2}$ conformed to a $\chi^{2}$ distribution with degrees of freedom equal to the number of tests minus the number of factors. Demonstration of the $d_{\mathrm{ri}}^{2}$ index in a large sample of clinical cases was also provided. Findings highlight the potential utility of the $d_{\mathrm{ri}}^{2}$ index as an adjunct to PVTs, offering clinicians an additional method to evaluate WAIS-IV test profiles and improve the accuracy of neuropsychological evaluations.

## Introduction

Approximately 500,000 neuropsychological examinations are administered each year in the United States (Bilder & Reise, 2019), with the Wechsler Adult Intelligence Scale Fourth Edition (WAIS-IV; Wechsler, 2008a) being one of the most widely used (Camara et al., 2000; Rabin et al., 2005) and well-validated (e.g., Bowdenet al., 2011; Climie & Rostad, 2011; Nelson et al., 2013) tests to examine both general and specific cognitive abilities in adults.

In recognition of the multiple biases that can potentially vitiate the interpretation of a WAIS-IV (e.g., response bias, malingering, inattention, lack of effort), a central task for a clinical neuropsychologist is to judge, for a particular patient, whether a protocol provides a credible, interpretable, or otherwise “valid” measure of the assumed underlying cognitive abilities. By far, the dominant approach to making such judgments relies on performance validity tests (PVTs; Lippa, 2017; Rickards et al., 2018). Thus, prior to introducing our model-based person fit approach to evaluating the interpretability of WAIS-IV profiles, we first review PVTs and several other indicators of response aberrancy.

### PVTs and the Evaluation of WAIS-IV Protocols

Sweet et al. (2021) noted, “In order to provide bases for diagnoses and interpretations, the current consensus is that all clinical and forensic evaluations must proactively address the degree to which results of neuropsychological and psychological testing are valid” (p. 1053). Heilbronner et al. (2009) define an invalid test as those that “(1) are not fully explained by brain dysfunction, (2) are not reasonably attributable to variables that may in some instances moderate (e.g., education, age) or may in some instances confound (e.g., fatigue, psychological conditions) performances on ability tests, and (3) are significantly worse than, or at least different in degree or pattern from, performance known to reflect genuine brain-based disturbances in neuropsychological abilities” (p. 1100).

Accordingly, over the last 30 years, considerable research has been dedicated to the development and evaluation of PVTs (Larrabee, 2012; Leonhard, 2023; Lippa, 2017; Sweet et al., 2021). Although PVTs were used historically for the detection of malingering (Leonhard & Leonhard, 2024), response bias, or poor effort (Jasinski et al., 2011), recent formulations describe PVTs as providing methods to judge whether the entire neuropsychological examination is credible, trustworthy, or, as most commonly stated, a valid reflection of cognitive abilities (Greher & Wodushek, 2017).

The two major classes of PVTs are stand-alone (tests developed to assess validity without an intention to measure abilities on other neuropsychological constructs), and embedded (tests designed to test Neuropsychology abilities, but then usually cross-validated with respect to other PVTs to identify scores so low that these are not credible). An example of a stand-alone PVT is The Test of Memory Malingering (TOMM; Tombaugh & Tombaugh, 1996), where each item is presented in forced-choice format, and the respondent is asked to identify which of two stimuli was previously presented. Responses are compared to chance level (50%) or norms for various clinical conditions (Martin et al., 2020). An example of an embedded PVT is the Reliable Digit Span (RDS), which is based on responses to the WAIS-IV Digit Span (DS) subtest (Jasinski et al., 2011). The RDS is the longest number of digits forward in which both trials of that length were correct, and the longest number of digits backward in which both trials of that length were correct. RDS scores less than 6 or 7 are common cutoff values for determining validity (Zenisek et al., 2016). The key idea for either the TOMM or RDS is that poor performance may not simply reflect cognitive deficits, but rather other behaviors or conditions that invalidate the test score or protocol.

### Discrepancy Analysis and the Mahalanobis Distance on the WAIS-IV

PVTs were originally designed to detect test performance so uncommonly poor that it calls into question the validity of the neuropsychological evaluation and the subsequent validity of clinical conclusions. PVTs serve as broad indicators and are not directly linked to the psychometric model that underpins and supports the interpretation of specific cognitive measures. As a complement to PVTs, to identify unusual and potentially uninterpretable profiles on the WAIS-IV, we propose the application of a model-based *person fit* statistic called the residual distance for an individual ( $d_{\mathrm{ri}}^{2}$ ; Yuan & Hayashi, 2010; Yuan & Zhong, 2008; Yuan et al., 2004). Large $d_{\mathrm{ri}}^{2}$ values flag profiles of WAIS-IV performance that are inconsistent with the factor model assumed to underlie the interpretation of the WAIS-IV profile. In the original literature, $d_{\mathrm{ri}}^{2}$ is referred to as a “model outlier” statistic, and it was used as a case weight to obtain robust parameter estimates.

**Figure 1.**
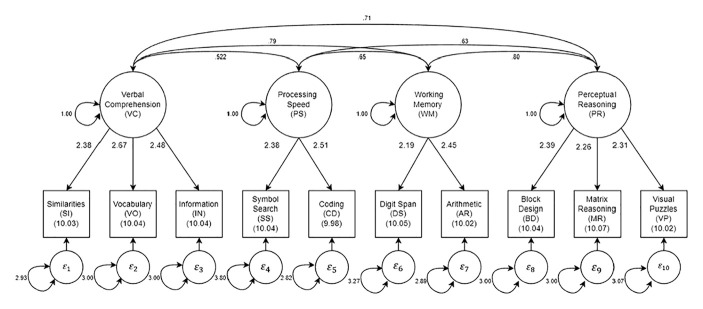
Parameter estimates for the four correlated factor solution in the standardization sample.

To understand the logic of a model-based person fit approach to identifying aberrant WAIS-IV profiles and how it differs from other traditional methods, we need to establish some context. First, the WAIS-IV contains 10 core subtests that assess specific cognitive abilities (see Figure 1). The 10 core subtests are typically aggregated into four “index scores” reflecting Verbal Comprehension (VC: Similarities [SI], Vocabulary [VO], Information [IN]), Perceptual Reasoning (PR: Block Design [BD], Matrix Reasoning [MR], Visual Puzzles [VP]), Working Memory (WM: DS, Arithmetic [AR]), and Processing Speed (PS: Symbol Search [SS], Coding [CD]). Subtests measuring the same index construct (factor) are highly correlated, and the four index scores tend to be moderately correlated, which suggests the presence of a general cognitive factor.

It is important to note that the suggested order of test administration (BD, SI, DS, MR, VO, AR, SS, VP, IN, and CD) places potential time gaps between the subtests in a factor during which attentional or other state fluctuations could reasonably be expected to occur. For example, the gap may be 10 to 15 min between Similarities and Vocabulary, and then another 15 to 20 min before Information. Similarly, the gap between AR and DS can also be quite long. Importantly, it is not always the case that the practitioner administers tests in the recommended order, and it is sometimes the case that the practitioner may insert other tests into the sequence.

Second, given this well-accepted structure, it is reasonable to assume that a patient responding according to the model of cognitive abilities underlying the WAIS-IV should have subscale scores within each index dimension that are relatively consistent, and, to a lesser extent, subscale scores that are consistent across cognitive factors. When subtest or index scores display large discrepancies, this may reflect a unique configuration of cognitive abilities or may reflect a faulty, possibly uninterpretable protocol. In the original form of the WAIS (Wechsler–Bellevue), it was suggested that differences in scores of more than two are considered significant and may suggest an aberrant response profile (Wechsler, 1941).

There is a long history of examining such subscale or index score discrepancies on WAIS instruments (e.g., Glass et al., 2010; Silverstein, 1982). For example, a clinician can evaluate the statistical significance of differences between the WAIS-IV Index Scores, pairs of individual subtest scores, a single subtest score, and the average of subtest scores, and inter-subtest scatter, which is defined as the difference between the highest and lowest subtest scaled scores.^1^

Such discrepancy score analyses yield numerous, often unreliable comparisons (Glass et al., 2010) and are mainly used for profile analyses and diagnosis—the analyses of cognitive strengths and weaknesses—rather than as a direct index of a patient’s WAIS-IV profile interpretability or validity. However, as Huba (1985) pointed out, in the spirit of “less is more,” it is easy to develop a single overall index of how “unusual or unique” (p. 322) the pattern of scores is across multiple variables. Such an index may be of more value in identifying potentially uninterpretable profiles. Specifically, a Mahalanobis distance (MD; see Appendix) can be calculated as:

(1) $${\mathrm{MD}}^{2}={\left(X_{i}-\bar{X}\right)}^{\prime }S^{-1}\left(X_{i}-\bar{X}\right)$$

where $X_{i}$ is a vector of observed scores on manifest variables for an individual i . The remaining parameters represent sample characteristics from which observations are compared to where $\bar{X}$ is a vector of means on manifest variables calculated within a sample, and $S^{-1}$ is the inverse of the covariance matrix among the manifest variables measured in that sample. When the manifest variables are distributed as multivariate normal, MD^2^ is distributed as $\chi^{2}$ with degrees of freedom equal to the number of variables. MD values are influenced by both the profile scatter and elevation. Large MD values may indicate that individuals may come from populations that are poorly characterized by the testing norms.

Drawing on Huba’s work, Crawford et al. (2012) used an MD index to estimate the atypicality of a profile based on the four WAIS-IV index scores. More relevant to the present investigation, Crawford and Allan (1994) and Burgess (1991) developed MD measures for the 10 WAIS-IV subtests, rather than the 4 index scores. Relatedly, Elfadaly et al. (2016) provided technical treatment and an updated estimator of the MD. Regardless of estimation method, the purpose remains the same, namely, to identify a profile of subtest scores that is unusual given an assumed profile of normative and valid responding.

Given this background, we note that the $d_{\mathrm{ri}}^{2}$ approach to evaluating person fit on the WAIS-IV is similar to the MD approach for identifying unusual response patterns in Crawford and Allan (1994) and in Burgess (1991). Specifically, both approaches compare a patient’s response profile to a model of expected responding. However, the comparison models are very different. The MD approach is based on means and covariances of manifest variables derived from a normative or standardization sample. Large discrepancies indicate how discrepant an individual is from the centroid (means) of that multivariate distribution. Importantly, the resulting MD values are influenced by both profile scatter and elevation (i.e., distances from the means).

By contrast, in a model-based person fit approach, an individual’s response pattern is compared to an expected response pattern based on estimated factor scores that are derived from a well-fitting structural equation model (SEM). In model-based person fit using $d_{\mathrm{ri}}^{2}$ , large discrepancies—indicative of poor person fit—suggest that the WAIS-IV profile deviates from the psychometric measurement model underlying its interpretation. Consequently, $d_{\mathrm{ri}}^{2}$ is not confounded by profile elevation. Moreover, Yuan et al. (2004) argued that when evidence of a measure’s structural validity is strong, as it is for the WAIS-IV, $d_{\mathrm{ri}}^{2}$ is preferred over aberrancy indices based on the observed variable MD.

### Model-Based Person Fit: Developing a Comparison Model for the WAIS-IV

Model-based person fit statistics such as $d_{\mathrm{ri}}^{2}$ require a well-fitting model to establish the factor structure used to justify the interpretation of scores on a measure. Herein, we refer to such a model as a comparison model. The comparison model is simply an SEM that is assumed to represent valid responding. Therefore, it must provide a good fit to the data in a well-defined examinee population to provide a meaningful reference for the interpretation of person fit indices computed for new examinees.

The estimated comparison model for this study is an SEM with 4 correlated factors for the 10 WAIS-IV subtests based on standardization sample data (*N* = 2,200; Wechsler, 2008a). Descriptive statistics for this sample are provided in the WAIS-IV manual. The four factors and their respective indicators, as well as the estimated parameters, are shown in Table 1 and Figure 1. Previously, we reported on extensive confirmatory factor analyses of the WAIS-IV as well as several other neuropsychological measures (Bilder et al., 2023). In those analyses, both standardization and clinical samples were evaluated, and a second-order factor model (general cognitive function and four primary factors) was judged to have the best statistical fit to the WAIS-IV. Nevertheless, we now consider only a model with four correlated factors, which is a less constrained version of the second-order model.

**Table 1.** Results of Confirmatory Factor Analysis of 10 Core Subtests of the WAIS-IV in the Standardization Sample.

Variable	Intercept	Factor loadings				Residual
		VC	PR	WM	PS	Variance
Similarities	10.03	2.38				2.92
Vocabulary	10.04	2.67				1.85
Information	10.04	2.48				3.33
Block design	10.04		2.39			3.63
Matrix reasoning	10.07		2.26			4.47
Visual puzzles	10.02		2.31			4.07
Digit span	10.05			2.19		4.27
Arithmetic	10.02			2.45		2.89
Symbol search	10.04				2.38	3.80
Coding	9.98				2.51	2.82
Factor intercorrelations						
VC		1				
PR		0.71	1			
WM		0.79	0.80	1		
PS		0.52	0.63	0.65	1	

*Note.* All scales have a mean of 10 and a standard deviation of 3. *N* = 2,200. Model fit values: χ^2^(29) = 283.05, *p*≤ .001, RMSEA = 0.063, SRMR = 0.026, CFI = 0.977. VC = verbal comprehension; PR = perceptual reasoning; WM = working memory; PS = processing speed; WAIS-IV = Wechsler Adult Intelligence Scale Fourth Edition; RMSEA = root mean squared error of approximation; SRMR = standardized root mean residual; CFI = comparative fit index.

Estimating the correlated factor model serves two goals. First, the person fit software (see details below) we use cannot presently handle complex higher-order models such as a second-order or bifactor model. Second, statistical fit indices for the four correlated factor model were acceptable and very close to those for the slightly better fitting second-order model. Specifically, using the lavaan package (Rosseel, 2012) in R version 4.4.3 (R Core Team, 2020) and full information maximum likelihood estimation, the fit for the standardization sample was satisfactory with χ^2^(29) = 283.05, *p*≤ .001, root mean squared error of approximation (RMSEA = 0.063), standardized root mean residual (SRMR = 0.026), comparative fit index (CFI = 0.977), and Tucker-Lewis index (TLI = 0.965) all falling within conventions of adequate fit. Note that for the standardization sample, all subtest means were roughly 10 with standard deviations equal to 3. In turn, those means are treated as factor intercepts in the modeling of person fit (described below).

### Model-Based Person Fit: Calculating the Index

Once a comparison model has been established, it is possible to evaluate the consistency of an individual’s response profile with the SEM used to interpret test scores (e.g., Yuan et al., 2004). Although person fit statistics have been well researched in item response theory (IRT) modeling contexts (Meijer, 2003; Meijer & Sijtsma, 2001), they have only recently been proposed for factor analytic models (Bollen & Arminger, 1991; Ferrando, 2009, 2010; Mansolf & Reise, 2018; Reise et al., 2016). To our knowledge, these factor-analytic-based person fit indices have not been applied to identify atypical response patterns for any well-known neuropsychological test. As such, the present application represents a novel exploration of the use of model-based person fit on the WAIS-IV subtests.

Most relevant to the present investigation are factor analytic person fit indices developed by Yuan et al. (2004), Yuan and Zhong (2008, 2013), and Yuan and Hayashi (2010). The objective of model-based person fit is to quantify the distance between the expected response pattern based on the comparison model parameters and the factor score estimates, and a given observed response pattern. To do so, the Bartlett factor score must be calculated to assess each (i) individual’s standing on the latent factor(s) (Bartlett, 1937). This is given by:

(2) $$f_{i}={\left(\Lambda \prime {\Sigma_{\varepsilon }}^{-1}\Lambda \right)}^{-1}\Lambda \prime (\Sigma_{\varepsilon }{)}^{-1}(x_{i}-\mu )$$

where *f*_i_ is the ith individual’s vector of factor scores. To characterize the comparison model, Λ is a ( p×q ) matrix of factor loadings of p observed variables on q latent factors. $\Sigma_{\varepsilon }$ is a p×p matrix of unique factor variances and covariances, $x_{i}$ is a vector of observed scores, and μ is a vector of means on the observed variables in the population.

Residuals from this model, which quantify the distance between the model and response pattern conditional on factor score estimates, are defined as:

(3) $$e_{\mathrm{fi}}=\left[I-\Lambda {\left(\Lambda \prime {\Sigma_{\varepsilon }}^{-1}\Lambda \right)}^{-1}\Lambda \prime {\Sigma_{\varepsilon }}^{-1}\right](x_{i}-\mu )$$

where I is a (p×p) identity matrix. The covariance matrix of $e_{\mathrm{fi}}$ is then:

(4) $$\Omega =\Sigma_{\varepsilon }-\Lambda {\left(\Lambda \prime \Sigma_{\varepsilon }^{-1}\Lambda \right)}^{-1}\Lambda \prime$$

as given by Bollen and Arminger (1991). However, Ω is of rank p−1 and therefore cannot be directly used to calculate $d_{\mathrm{ri}}^{2}$ . Thus, a new matrix A must be created from the non-zero eigenvalues of Ω as columns to alleviate the issue of non-invertibility. A is then a p×q matrix with columns orthogonal to $\Sigma_{\varepsilon }^{-1}\Lambda .$ We now have all of the necessary elements to calculate the distance residual based person fit statistic, $d_{\mathrm{ri}}^{2}$ (Yuan & Zhong, 2008), given:

(5) $$d_{\mathrm{ri}}^{2}=\left(A\prime e_{\mathrm{fi}}\right)\prime {\left(A\prime \Omega A\right)}^{-1}(A\prime e_{\mathrm{fi}})$$

which measures how much of an outlier a given case is from the factor model.^2^ $d_{\mathrm{ri}}^{2}$ is sensitive to large subtest score discrepancies (inconsistencies) within constructs, but less so between constructs. A large $d_{\mathrm{ri}}^{2}$ indicates a response pattern that is inconsistent with the psychometric measurement model (i.e., the latent model structure) of the abilities assumed to underlie test performance. Stated differently, large $d_{\mathrm{ri}}^{2}$ values result from large differences between the observed pattern of subtest scores within each factor and the predicted subtest scores that are based on the factor score estimates and model parameters.

Assuming multivariate normality, under the null hypothesis of good model fit, $d_{\mathrm{ri}}^{2}$ is distributed as $\chi^{2}$ with degrees of freedom $p_{i}-q$ where $p_{i}$ is the number of subtests administered to individual i . The $d_{\mathrm{ri}}^{2}$ values can be requested from *lavaan* (Rosseel, 2012) using a model object that contains the estimated model parameters, effectively fixing all estimable parameters in the model. For example,

SEM_Model_Fit=lavaan(mod01,data=Data_Frame,estimator=′ML′,missing=′FIML′)

would fit the data (***Data_Frame***) to the model structure specified by ***mod01***, with all parameters fixed to the parameters estimated in the comparison sample. Then,

***ModelOut = lavPredict(SEM_Model_Fit, type = “resid”, method = “Bartlett”, mdist =TRUE)***

where ***SEM_Model_Fit*** is the fit of four correlated factor model (Table 1), ***“resid”*** requests the $d_{\mathrm{ri}}^{2}$ M-Distance, ***“Bartlett”*** requests Barlett factor score estimates, and ***mdist = TRUE*** tells lavaan to estimate a MD.

**Figure 2.**
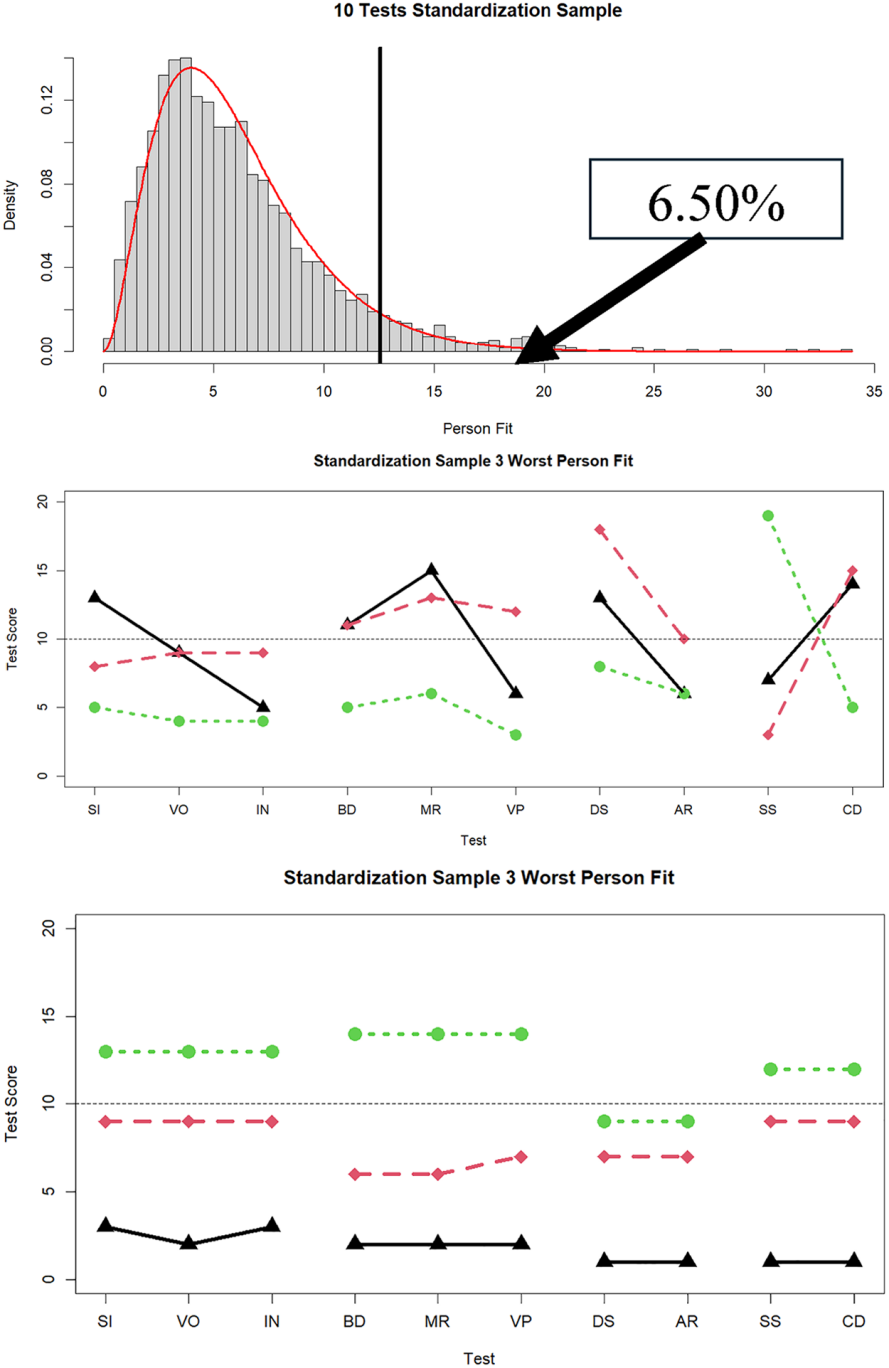
Distribution of $d_{\mathrm{ri}}^{2}$ in the standardization data (*N* = 2,200; % significant α = .05 in box), and three cases with the worst and best person fit in the standardization sample data. *Note.* SI = similarities; VO = vocabulary; IN = information; BD = block design; MR = matrix reasoning; VP = visual puzzles; DS = digit span; AR = arithmetic; SS = symbol search; CD = coding.

In the top panel of Figure 2, we display the distribution of $d_{\mathrm{ri}}^{2}$ in the WAIS-IV standardization sample (*N* = 2,200). Given that this sample was used to estimate the comparison model, and the model fits the data well, it is not surprising that the distribution of $d_{\mathrm{ri}}^{2}$ conforms well to a $\chi^{2}$ distribution with *df* = 6 (10 manifest variables minus 4 factors). However, the rejection rate at α=.05 is 6.5%. This is due to both violations of multivariate normality, and a few cases with relatively large misfit. In the middle panel of Figure 2 are the response patterns of the three individuals with the worst fit in the standardization sample ( $d_{\mathrm{ri}}^{2}$ = 33.59, 32.47, and 31.12, respectively). These individuals display profound variance in subtest scores within one or more factors. Specifically, the individual represented by the black line exhibits large within-factor discrepancies on all four factors; the person shown in red had large within-factor discrepancies on only the last two factors; and the person shown in green exhibited a very large discrepancy on the fourth factor. In the bottom panel of Figure 2 are the three best fitting individuals ( $d_{\mathrm{ri}}^{2}$ = 0.03, 0.18, and 0.24, respectively) who differed in elevation but showed within-factor profiles that were nearly flat.

## Present Research

As noted, person fit statistics based on factor analytic models are relatively new and have seen little application to clinical data. With this in mind, we conducted two studies. In the first, based on the comparison SEM estimated on the WAIS-IV standardization sample (Table 1 and Figure 1), we conducted a Monte Carlo simulation. The two main objectives of this simulation were (a) to determine whether $d_{\mathrm{ri}}^{2}$ is computable and (b) adheres to its theoretical sampling distribution, $\chi^{2}$ with degrees of freedom equal to $p_{i\;}$ −*q*, under varying degrees of missing WAIS-IV subtest data. This is critically important because, in clinical assessment, few patients are administered the entire battery of 10 subtests, and the functioning of $d_{\mathrm{ri}}^{2}$ has not yet been established either using the WAIS-IV model or in the presence of missing subtests.

There is good reason to be skeptical of the proposed sampling distribution for $d_{\mathrm{ri}}^{2}$ in the present context. For example, in the presence of missing subtests, factor scores may not be computable, or, if computable, may be extremely biased with large standard errors. In turn, this could severely impact both the calculation of $d_{\mathrm{ri}}^{2}$ (which cannot be computed when a factor score estimate is missing) or may severely bias its values.

In the second study, we applied the person fit statistic in a large sample of clinical cases. This second study is more for exploratory and demonstration purposes and illustrates different types of patterns of poor person fit, rather than hypothesis testing per se. Nevertheless, empirically, we tracked both the distribution of $d_{\mathrm{ri}}^{2}$ (and percent rejected at α=.05) and the percent of cases for which $d_{\mathrm{ri}}^{2}$ was computable under different degrees of missing subtests. Finally, this analysis also allowed us to informally review the neuropsychological profiles of individuals with high and low $d_{\mathrm{ri}}^{2}$ values.

## Methods and Results

### Study 1: Monte Carlo Simulation

To obtain a realistic view of WAIS-IV administration in practice, our first step was to examine the pattern of missing data among the individuals who were enrolled in the National Neuropsychology Network (https://www.nnn.ucla.edu/) project and were administered at least one subtest of the WAIS-IV (*N* = 4,823). Patient demographic characteristics are shown in Table 2. We note that, when a patient does not receive all 10 WAIS-IV subtests, the missing tests are typically not missing at random but rather due to test protocol variations across participating sites. Consequently, some subtests are almost always administered and others much less so. The subtests that are administered are decided by the clinician based on what they believe necessary for the neuropsychological evaluation.

**Table 2.** Descriptive Statistics for Clinical Sample.

Characteristic	*N*	Mean (*SD*)
Age	4,823	55.49 (18.18)
Education years	3,565	15.36 (5.69)
Sex		Percent
Male	2,288	47.46
Female	2,526	52.4
Unknown	3	0.06
Prefer not to answer	4	0.08
Race		
White	3,632	75.31
Black	701	14.53
Asian	133	2.76
Native Hawaiian/other Pacific Islander	3	0.06
Native American/Alaskan native	12	0.25
Other	113	2.34
Unknown	195	4.04
Prefer not to answer/declined to specify	32	0.66
Ethnicity		
Hispanic or Latino	118	2.45
Not Hispanic or Latino	4,500	93.30
Unknown/missing	203	4.21

The SEM shown in Table 1 and Figure 1 was used as the basis for a Monte Carlo simulation. In the design of the simulation, cases where the number of WAIS-IV subtests administered was five or less (about 33% of the clinical sample) were not considered because person fit could not be computed when there were four or fewer tests, and person fit could not reliably be computed when only five tests were administered.

Next, using the clinical data, the sample was separated into subgroups based on the number of tests administered, ranging from 6 to 10. One hundred thousand cases based on the model in Figure 1 and Table 1 were simulated for each subtest condition. Then, for each condition and each simulated subject, a missing data pattern from the relevant clinical data set was randomly sampled and assigned to a simulated case. Thus, the simulated data matched, probabilistically, the missing data patterns at each number of tests administered level (i.e., 6–10 subtests).

For each level of the number of subtests administered, the expectation was that the person fit statistic should be distributed as $\chi^{2}$ with mean equal to the degrees of freedom (*p* *−* *q*) and variance equal to 2(p−q) . In the middle panel of Table 3, the computable *N* and % (computable) columns show that when 8, 9, or 10 WAIS-IV subscales were administered, $d_{\mathrm{ri}}^{2}$ was almost always computable; however, when only 6 or 7 subtests were administered, $d_{\mathrm{ri}}^{2}$ could not be computed approximately 25% of the time. This occurred mostly when there were missing subtest scores for a factor that has only two indicators, and thus, no factor score could be estimated. Nevertheless, for computable cases of $d_{\mathrm{ri}}^{2}$ , the mean and variance of the sampling distribution appeared to conform to its theoretical value at all levels of the number of subtests administered. Moreover, the percentage rejected at α=.05 was accurate under these simulated conditions.

**Table 3.** Distribution of $d_{\mathrm{ri}}^{2}$ in Standardization Sample Data, Simulated Data, and Clinical Data as a Function of Number of Tests Administered.

Number of tests (*df*)	Starting *N*	Computable *N*	Computable (%)	Mean $\;d_{\mathrm{ri}}^{2}$	Variance $d_{\mathrm{ri}}^{2}$	% Significant α=.05
Standardization sample						
10 (6)	2,200	2,200	100	6.00	15.89	6.5
Simulated sample						
10 (6)	100,000	100,000	100	6.00	12.04	5.05
9 (5)	100,000	100,000	100	5.00	9.98	5.00
8 (4)	100,000	98,227	98.23	4.00	7.98	5.01
7 (3)	100,000	74,780	74.78	3.01	6.05	5.01
6 (2)	100,000	79,211	79.21	2.00	4.00	5.02
Clinical sample						
10 (6)	188	188	100	6.77	19.63	12.76
9 (5)	175	175	100	4.92	10.68	4.00
8 (4)	459	451	98.25	4.41	10.96	7.53
7 (3)	424	318	75.00	3.16	8.35	7.23
6 (2)	1,967	1,559	79.25	1.98	5.49	5.51

*Note. df* = degrees of freedom, *p* − *q*.

### Study 2: Person Fit in a Clinical Sample

Having established a statistical comparison model for valid responding, we now turn to the estimation of person fit within a clinical sample, as described above. The calculation of person fit in the clinical sample followed the same procedure as described earlier using lavaan. The key difference is that the clinical sample did not contribute to the model; instead, the model has all parameters fixed to those estimated in the comparison sample, allowing person fit to be interpreted within the desired metric of model-response pattern discrepancy.

**Figure 3.**
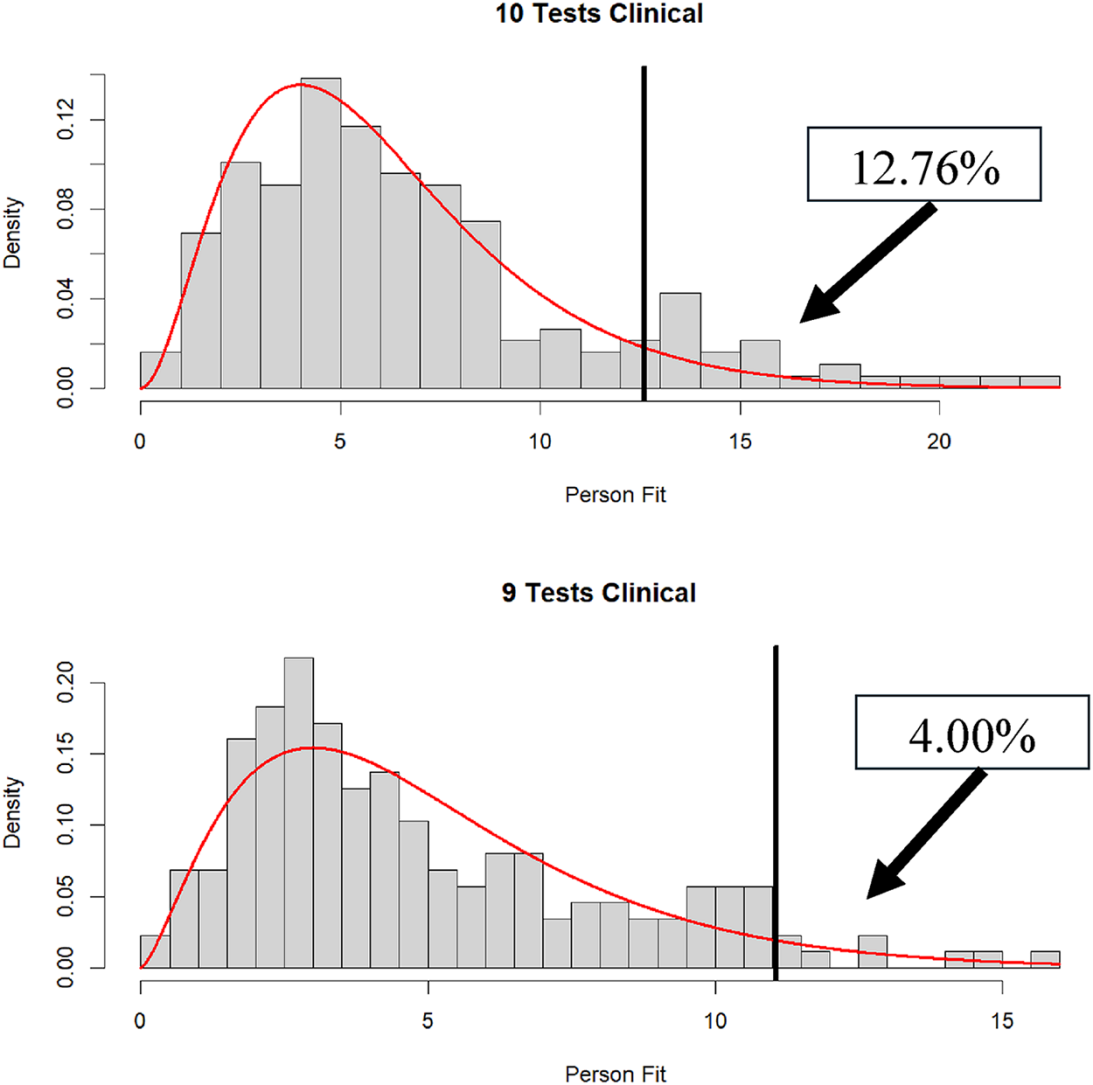
Distribution of $d_{\mathrm{ri}}^{2}$ in clinical data when 10 (*N* = 188) and 9 (*N* = 175) subtests are administered (% significant α = .05 in box).

**Figure 4. fig4-00131644251339444:**
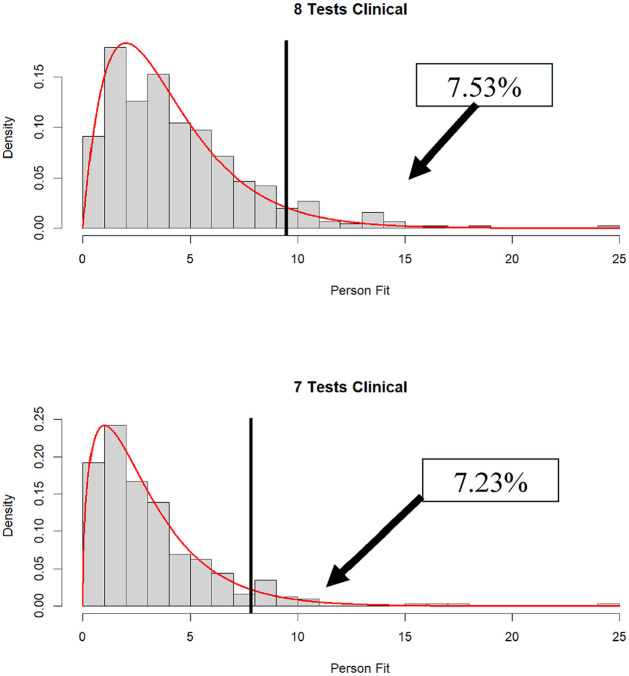
Distribution of $d_{\mathrm{ri}}^{2}$ in clinical data when eight (*N* = 451) and seven (*N* = 318) subtests are administered (% significant α = .05 in box).

The results for individuals receiving between 6 and 10 subtests are shown in the bottom panel of Table 3. Anyone who received fewer than six WAIS-IV subtests was excluded from the analyses. The results show that a relatively large number of participants were administered six WAIS-IV subtests, with relatively few participants being administered 9 or 10 subtests. Going from 6 to 10 tests administered, the percentages of $d_{\mathrm{ri}}^{2}$ that were computable were as follows: 79%, 75%, 98%, 100%, and 100%, respectively. This is consistent with the simulated results, with large amounts of missing values leading to inability to calculate $d_{\mathrm{ri}}^{2}$ values around 23% of the time. Interestingly, the clinical sample tended to have approximately the same mean $d_{\mathrm{ri}}^{2}$ values as in the simulated data, but the variances were larger. Consistent with this finding, the rejection rates for the clinical sample were higher than expected by chance at α = .05 (except for the nine-subtest condition). In fact, almost 13% were statistically significant at α = .05 in the 10-subtest condition. That said, for the remaining conditions, rejection rates were not far from their nominal values, implying that patients, for the most part, were responding in accordance with the model (see Figures 3 [Fig fig4-00131644251339444]–5 for plots of the distributions of $d_{\mathrm{ri}}^{2}$ in the clinical data).

**Figure 5.**
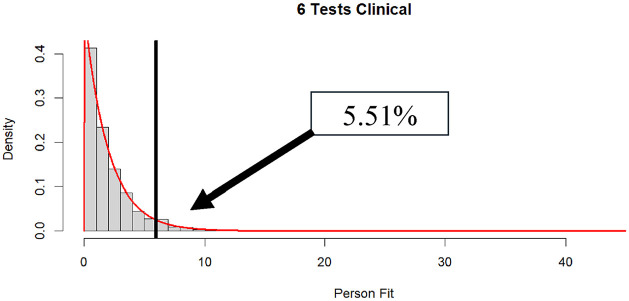
Distribution of $d_{\mathrm{ri}}^{2}$ in clinical data when six (*N* = 1,559) tests are administered (% significant α = .05 in box).

**Figure 6.**
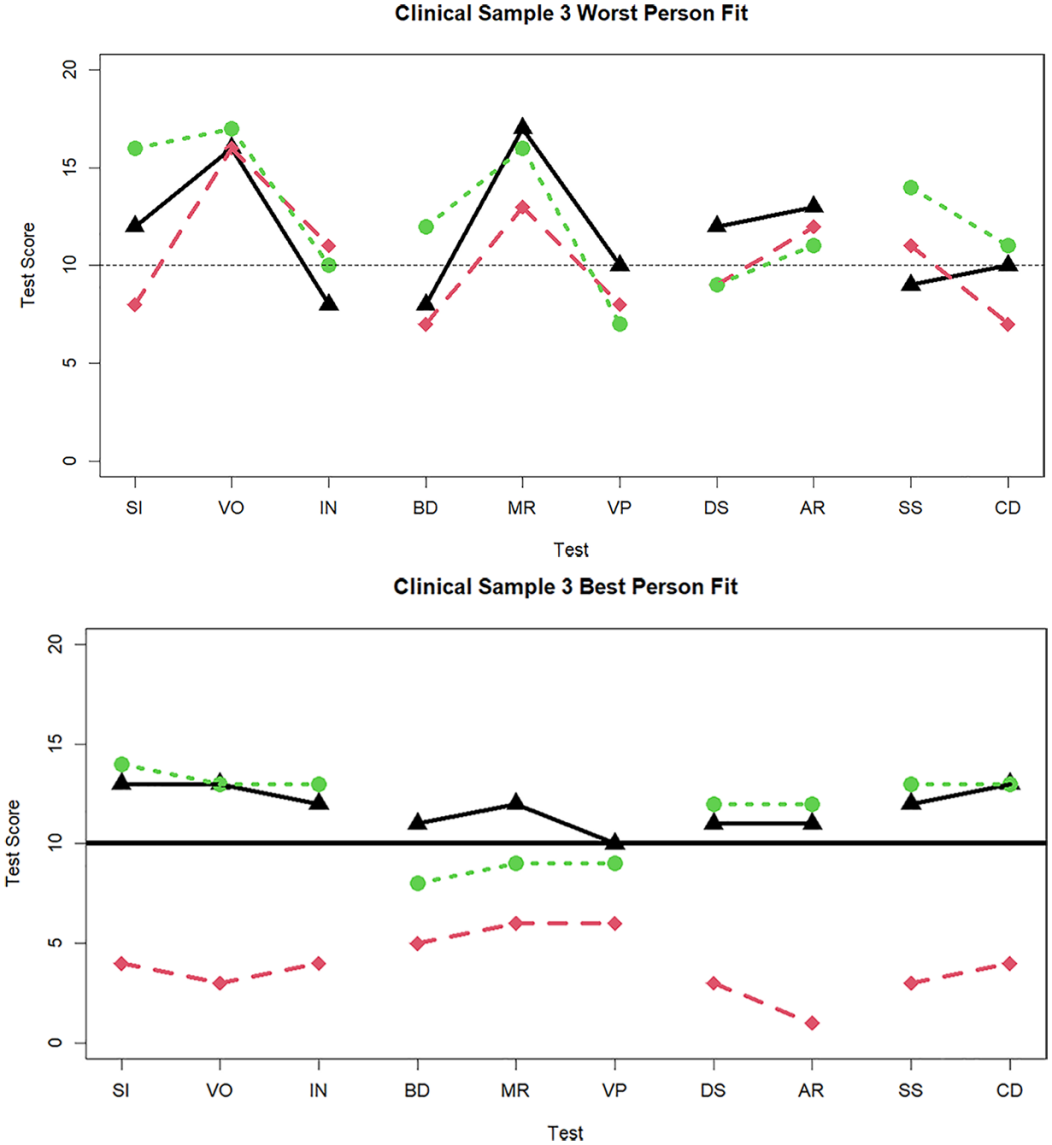
Three cases with the worst and best person fit in the clinical sample data when 10 tests are administered. *Note*. SI = similarities; VO = vocabulary; IN = information; BD = block design; MR = matrix reasoning; VP = visual puzzles; DS = digit span; AR = arithmetic; SS = symbol search; CD = coding.

Finally, for illustrative purposes and to parallel Figure 2 (standardization sample), in Figure 6 we display profiles for the three best-fitting and worst-fitting individuals from the clinical sample, within the 10-subtest condition. For these individuals with large $d_{\mathrm{ri}}^{2}$ (22.89, 21.47, 20.91), interpreting the source of poor fit appears straightforward. Specifically, it appears there was a spike for one subtest (i.e., VO and MR) within each of the first two factors, relative to the other two indicators. In the bottom panel, much like in the standardization sample, the best-fitting profiles revealed relatively flat profiles within and between factors ( $d_{\mathrm{ri}}^{2}$ = 0.57, 0.62, 0.79). These three cases also differ in elevation, demonstrating how $d_{\mathrm{ri}}^{2}$ is not sensitive to overall levels of the latent factors, unlike previously proposed MD methods (Burgess, 1991; Crawford & Allan, 1994).

**Figure 7.**
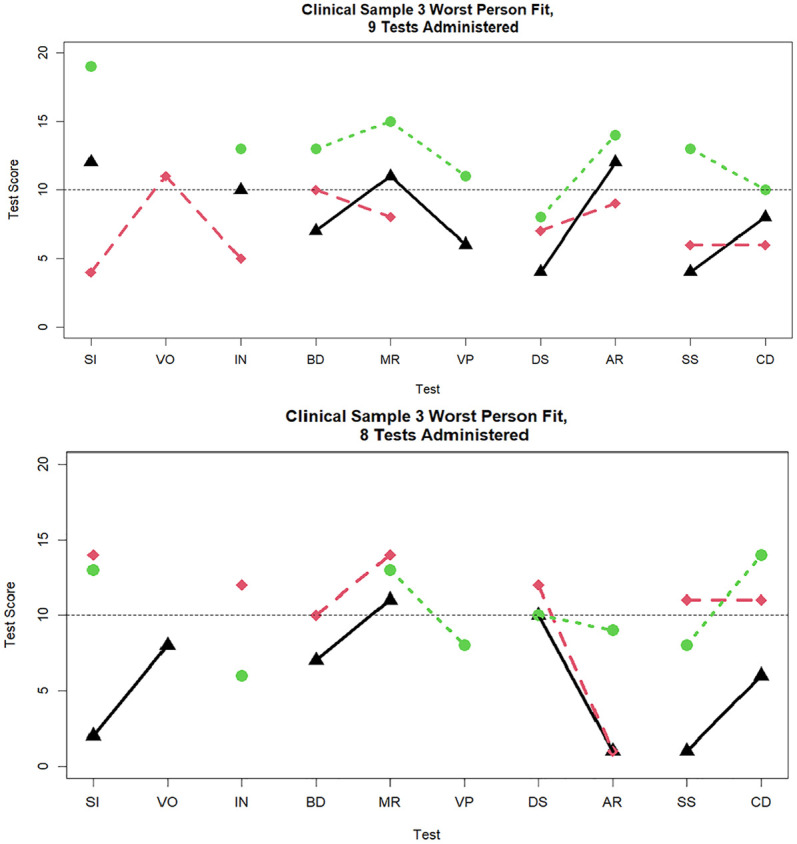
Three cases with the worst person fit in the clinical sample data when nine and eight tests are administered. *Note.* SI = similarities; VO = vocabulary; IN = information; BD = block design; MR = matrix reasoning; VP = visual puzzles; DS = digit span; AR = arithmetic; SS = symbol search; CD = coding.

**Figure 8.**
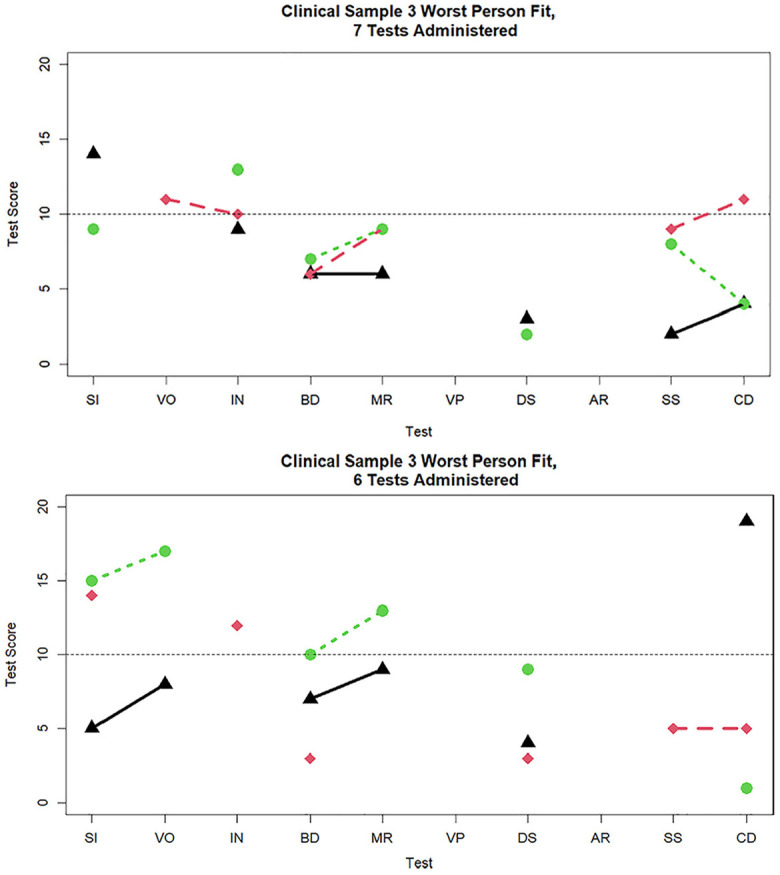
Three cases with the worst person fit in the clinical sample data when seven and six tests are administered. *Note.* SI = similarities; VO = vocabulary; IN = information; BD = block design; MR = matrix reasoning; VP = visual puzzles; DS = digit span; AR = arithmetic; SS = symbol search; CD = coding.

Figures 7 and 8 display the three worst-fitting profiles in the clinical sample for the nine ( $d_{\mathrm{ri}}^{2}$ = 15.69, 14.88, 14.31), eight ( $d_{\mathrm{ri}}^{2}$ = 24.86, 18.05, 16.21), seven ( $d_{\mathrm{ri}}^{2}$ = 24.25, 17.55, 16.07), and six ( $d_{\mathrm{ri}}^{2}$ = 44.88, 18.71, 18.68) subtests administered, respectively. To provide a more detailed picture, in Table 4, we display the subtest scores for the five worst-fitting individuals at each number of subtests administered condition. It can be seen that $d_{\mathrm{ri}}^{2}$ is sensitive to large discrepancies between observed and expected responses within one or more factors. Stated differently, poor person fit is caused by very low subtest scores (e.g., scores of 1 or 2) on one or two subtests and then relatively high scores on other subtests (e.g., 15 or 16). For example, among persons who received 10 subtests, the case with misfit of $d_{\mathrm{ri}}^{2}=$ 20.90, had relatively high VO Index scores of 16 on SI and 17 on VO, but then a much lower 10 on IN, a high Perceptual Reasoning Index scores of 12 on BD and 16 on MR, but then a very low 7 on VP, exhibiting within-factor subtest differences on both of the first two factors. Among persons who received six subtests, the case with the largest misfit of $d_{\mathrm{ri}}^{2}=$ 44.80 had a very large PS Index score of 15 on SS, but only a 1 on CD; the next case, with misfit of $d_{\mathrm{ri}}^{2}=$ 18.71, had the reversed pattern of scores, with a very low 3 on SS, but substantially higher score of 14 on CD. So, both of these latter cases showed very large differences in scores on the two indicators of PS.

**Table 4.** Subscale Scores for the Five Worst Fitting Individuals Within Each Number of Tests Administered Conditions.

Verbal comp.			Perceptual reas.			Working mem.		Proc. speed		*N* test	$d_{\mathrm{ri}}^{2}$
SI	VO	IN	BD	MR	VP	DS	AR	SS	CD		
12	16	8	8	17	10	12	13	9	10	10	22.80
8	16	11	7	13	8	9	12	11	7	10	21.47
16	17	10	12	16	7	9	11	14	11	10	20.90
9	18	14	13	9	12	11	12	12	8	10	19.60
9	12	13	9	12	11	19	9	12	12	10	18.71
12	-	10	7	11	6	4	12	4	8	9	15.79
4	11	5	10	8	-	7	9	6	6	9	14.80
19	-	13	13	15	11	8	14	13	10	9	14.31
15	12	8	-	12	15	17	14	14	18	9	12.89
16	10	-	11	12	7	12	10	8	10	9	12.79
2	8	-	7	11	-	10	1	1	6	8	24.86
14	-	12	10	14	-	12	1	11	11	8	18.05
13	-	6	-	13	8	10	9	8	14	8	16.21
11	15	-	7	15	11	7	13	6	-	8	15.62
14	-	13	10	13	-	15	5	9	9	8	14.90
10	-	7	13	4	-	5	-	3	12	7	24.25
12	-	10	1	11	-	8	-	10	16	7	17.54
11	-	11	11	16	-	6	-	2	11	7	16.07
14	15	-	7	12	-	9	-	1	10	7	15.99
15	-	10	9	14	-	13	-	11	16	7	10.87
16	-	6	11	-	-	12	-	15	1	6	44.88
12	-	13	9	-	-	10	-	3	14	6	18.71
5	14	-	9	13	-	11	-	-	9	6	18.68
4	12	-	9	12	-	5	-	-	3	6	14.89
3	11	-	-	7	6	11	-	1	-	6	14.27

*Note.* Verbal comp. = verbal comprehension index; perceptual reas. = perceptual reasoning index; working mem. = working memory index; proc. speed = processing speed index; SI = similarities; VO = vocabulary; IN = information; BD = block design; MR = matrix reasoning; VP = visual puzzles; DS = digit span; AR = arithmetic; SS = symbol search; CD = coding; *N* test = number of tests administered; $d_{\mathrm{ri}}^{2}$ = distance residual. Dash (-) indicates that a subtest was not administered

## Discussion

The WAIS-IV is a core component of many neuropsychological batteries. As individual profiles are used to formulate diagnosis and treatment decisions, it is essential to evaluate whether any individual WAIS-IV profile is interpretable as a valid reflection of cognitive abilities. In the present research, we briefly reviewed the roles of PVTs, discrepancy indices, and the MDs in judging the interpretability of a WAIS-IV assessment. As an alternative to these indices, we promoted a model-based person fit statistic, $d_{\mathrm{ri}}^{2}$ , based on the work of Yuan and Hayashi (2010), Yuan and Zhong (2008, 2013), and Yuan et al. (2004) that indicates the degree to which a patient’s pattern of subscale scores is consistent with the measurement model used to interpret those scores.

We then used a Monte Carlo simulation to evaluate whether the hypothesized $\chi^{2}$ sampling distribution held under different degrees of missing subscale score data. Results showed that if the number of subtests was six or more, the assumed sampling distribution closely approximated $\chi^{2}$ with degrees of freedom equal to the number of observed test scores minus four (the number of factors). However, when the number of subtests was eight or more was $d_{\mathrm{ri}}^{2}$ almost always computable; with six or seven subtests administered, approximately 25% of the time $\;d_{\mathrm{ri}}^{2}$ was not computable.

In our application to clinical data, we found that poor person fit was much more pronounced than expected in individuals with data for all 10 subtests. This could mean that these individuals are fundamentally different in some way from those in a non-clinical sample or that the power to detect misfit is maximized under this condition. By contrast, the proportion of poor persons fit when fewer than 10 subtests were administered did not depart as much from the expected value. This could be due to having fewer ways to be aberrant when fewer tests are administered. In the following, we further consider the interpretation of model-based person fit indices such as $d_{\mathrm{ri}}^{2}$ in clinical data.

### Interpreting Model-Based Person Fit Indices

Profile variability indices such as the MD (Crawford & Allan, 1994) represent the probability that an individual’s profile is a random sample from a population with a known mean vector and covariance matrix. Large values are associated with unlikely response patterns, due to either extremity or variability in subtests. By contrast, indices such as $d_{\mathrm{ri}}^{2}$ describe how likely a profile of WAIS-IV subscale scores is relative to predicted responses based on a specific SEM, in our case, the estimated parameters of a model with four correlated factors. This is a critical distinction: $d_{\mathrm{ri}}^{2}$ values are relatively immune to overall profile elevation and discrepancies between the four WAIS-IV factors, MDs based on observed subtest scores are not. In distinction to the standard MD measure, $d_{\mathrm{ri}}^{2}$ values are sensitive only to discrepant scores across subtests within factors.

Understanding the proper statistical meaning of $d_{\mathrm{ri}}^{2}$ is important, but equally important is understanding what it means about the interpretation of the WAIS-IV profile. In IRT, numerous person-fit statistics have been developed and are referred to by many names, including *test score caution indices* (Tatsuoka, 1984), *test score appropriateness* indices (Drasgow et al., 1987), as well as *test score scalability* indices (Ferrando, 2007; Reise & Flannery, 1996). These names all imply that the test scores associated with poorly fitting profiles should, at the least, be treated with caution and may not reflect the intended construct.

In terms of SEM person fit indices, earlier it was noted that $d_{\mathrm{ri}}^{2}$ was originally referred to as a “model outlier” index—a case that made the model fit worse. In our view, estimating a well-fitting SEM is an important part of establishing the (factor) structural validity of a measure for a given population. Thus, when an individual has a large and significant $d_{\mathrm{ri}}^{2}$ , this implies that the structural model operating for the population may not be governing the responses for a particular individual. In other words, large $d_{\mathrm{ri}}^{2}$ implies that the subscale scores are not good indicators of the cognitive abilities assumed to underlie test performance. It is in this sense that we can view $d_{\mathrm{ri}}^{2}$ as a complement to a PVT, as we *might*, in conjunction with other evidence, interpret a significant $d_{\mathrm{ri}}^{2}$ value as reflecting an invalid or uninterpretable protocol. This interpretation is consistent with Ferrando et al. (2016), who stated, “if a response pattern is not well explained by the model, there is no guarantee that the score assigned to this pattern will adequately reflect the ‘true’ trait level of the individual” (p. 2).

On the other hand, the finding of a significant level of person misfit is not an end, but an opportunity for investigation. Poor person fit may be due to any number of issues. One set of issues concerns what might be considered essentially artifactual bases associated with the person being evaluated (Schretlen et al., 2008). Just as with many PVTs, persons being tested may perform much worse than expected on particular subtests for many reasons. The aforementioned TOMM was developed as a measure of malingering; TOMM items tend to be so easy that one plausible interpretation of a low score is that the person being assessed malingered (i.e., attempted to underperform) or was not motivated to perform well. Alternatively, the basis for a large discrepancy between scores on subtests that are expected to yield similar scores could be due to inattention or fatigue.^3^ Fatigue may be a reasonable assumption, particularly in a clinical sample, especially if the testing time is lengthy. If an individual’s mind wanders during the testing session, a simple lack of attention to the task might have occurred. Examiner factors such as mishandling the stopwatch, iPad, personal biases about the examinee, and administration and scoring errors, and administration errors should also be taken into account as potential sources of poor person fit.

A second set of issues related to poor person fit concerns conjectures regarding substantive bases for poor fit. Substantive interpretation of poor person fit is not a novel proposal. It may be the case that individuals who provide a poor fit to an established model may provide information on personality, behavior, or other elements not captured by the model. Person fit measures not only provide a more comprehensive analysis of model-data discrepancies but also allow users to flag anomalous response profiles, which necessitate closer examination (Yuan & Hayashi, 2010).

Consistent with this substantively meaningful view, using IRT methods, many studies have attempted to understand the substantive sources of poor person fit (Meijer, 2003; Meijer et al., 2016). Conijn et al. (2015) reported that person misfit was related to the degree of overall psychopathology, particularly with psychotic and somatoform disorders. Moreover, Wanders et al. (2018) conducted interviews to explore the underlying cause of misfit for individuals responding to an inventory of depression symptomology. They found that, for 19 of 20 patients, poor person fit could be attributed to complex comorbidities, somatic complaints (e.g., endorsing severe symptoms but not mild symptoms, which, in turn, makes them seem higher in depression, when in fact, they only have somatic complaints), and neurological abnormalities.

In terms of the WAIS-IV, when neuropsychological performance does not align with a well-established statistical model, poor person fit may reflect clinical conditions rather than a lack of effort or engagement, or administrator error. As described in Loring and Goldstein (2019), large score discrepancies and, therefore, misfit may be related to patient pathology, such that specific patterns of misfit may be due to cognitive dysfunction. Indeed, in our informal review of the neuropsychological records of individuals who had poor fit, it appears that some misfit reflects clinically meaningful score differences that fit well with demonstrated neuropathology, while others appear to reflect inconsistent performance, more likely associated with variable effort or task engagement.

### Limitations

One limitation of $d_{\mathrm{ri}}^{2}$ as applied to the WAIS-IV is that it appears applicable only for individuals who receive at least six subtests. In our clinical sample, this represented approximately 66% of all individuals who received at least one WAIS-IV subtest, so misfit values could not be computed for a full one-third of our sample. Another potential limitation of our present work is the lack of research on the power of the $d_{\mathrm{ri}}^{2}$ statistic or any other measure to detect responses that are misaligned with the model. We demonstrated that, under the presence of missing data, $d_{\mathrm{ri}}^{2}$ adhered to a $\chi^{2}$ distribution as expected in simulated data. However, the fact that, in clinical data, those who received 10 subtests had much higher rejection rates than nominal levels, but persons with between 6 and 9 subtest scores available had only mildly elevated rejection rates, suggests that the identification of response aberrance may be dependent on the amount of data available. That is, with more information (i.e., less missing data), response aberrance may be more easily detected.

Finally, it is important to keep in mind that in the WAIS-IV, two of the factors have only three indicators, and the remaining two factors have only two indicators. Despite the relatively high loadings, factor score estimates have relatively large standard errors, and if one or two tests are missing, the factor score estimate can be wildly biased with a much larger standard error. Moreover, if all indicators of a factor are missing, the factor score cannot be estimated. Although these concerns did not appear to negatively impact the functioning of $d_{\mathrm{ri}}^{2}$ in the simulations, it must be recognized that the factor score estimates determine the expected or predicted subscale score and thus the residual. Further research is clearly needed on this issue.

### Summary

We proposed a novel application of factor-analytic-based person fit indices to the evaluation of WAIS-IV protocols. It appears that the hypothesized sampling distribution for the person fit statistic holds reasonably well when the number of tests administered is at least six. We view model-based person fit indices as a complement to traditional PVTs, which are more aligned with identifying malingering and other forms of faultiness than aberrant patterns of subscale scores. Model-based person fit can also be viewed, much like the MD statistics, as a way of summarizing the numerous possible discrepancies between scale scores. The major caveat is that $d_{\mathrm{ri}}^{2}$ is only sensitive to within-factor subtest discrepancies, but not between-factor mean differences. Yet, it is important to note that between-factor discrepancies are much more likely to reflect specific pathologies than be prognostic of poor person fit or profile invalidity. One major advantage of model-based person fit is that it is rooted in a well-fitting psychometric model, which, in turn, is used to justify the interpretation of scale scores as reflecting underlying factors. As we noted previously, when a response pattern is inconsistent with the model, it is hard to argue that the scores are a reflection of the assumed underlying abilities. A critically important future line of research is to explore the substantive meaning of different types of poor person fit. Examination of the entire neuropsychological profile and history is one post hoc method of better understanding the source of poor person fit.

## Figures and Tables

**Figure A1.**
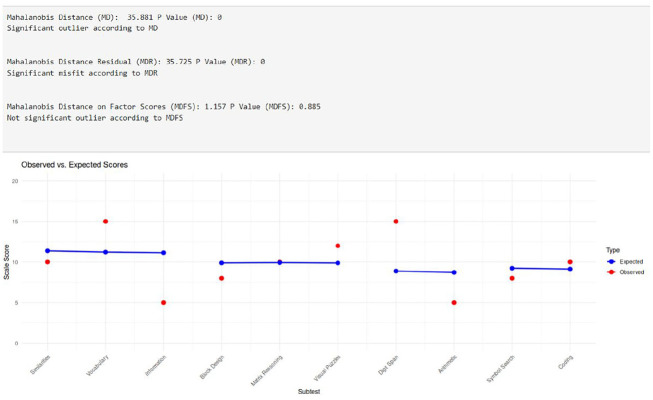
Shiny App Example of a large MDR. *Note.* MDR = Mahalanobis distance residual.

**Figure A2.**
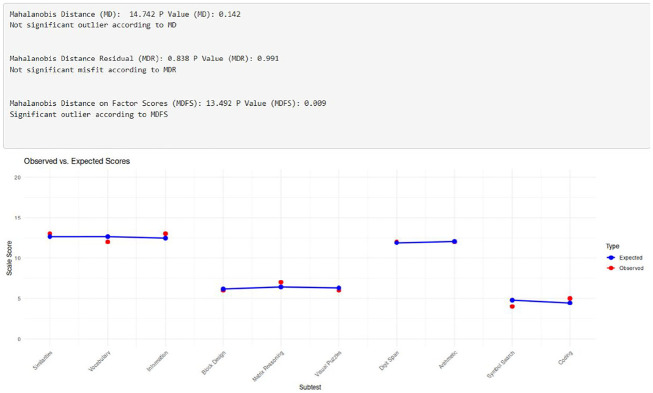
Shiny App Example of a large MDFS. *Note.* MDFS = Mahalanobis distance of the four factor scores.

## Appendix

### Using the Shiny App

A shiny app is available at https://wais-iv-pfit.psych.ucla.edu/ that provides three indices. First is the MD based on the WAIS-IV standardization sample. This index is distributed as $\chi^{2}$ with *df* equal to the number of core subtests administered. The *p* value reflects the probability that the case is a random sample from the standardization population. Second is the $d_{\mathrm{ri}}^{2}$ index, called the Mahalanobis distance residual (MDR). This is distributed as $\chi^{2}$ on *df* equal to (number of core subtests administered–4). The *p* value is interpreted as the probability of the observed response pattern given the expected pattern under the four-factor model.

Finally, the third index is the Mahalanobis distance of the four factor scores (MDFS). This index reflects the elevation and variability of the four-factor score estimates and is a complement to $d_{\mathrm{ri}}^{2}$ . This index is distributed as $\chi^{2}$ on 4 *df*. The *p* value reflects how likely the factor scores are a random sample from the population Φ matrix (correlation between the factors).

Below, we use the shiny app to illustrate extremes on MDR and MDFS (see Appendix Figures A1 and A2 respectively). The red lines are the observed subtest scores, and the blue lines are the expected subtest scores based on the model.

## Notes

1. WAIS-IV Technical and Interpretive Manual, Wechsler (2008b), pp. 53–55; appendix C tables; and WAIS-IV Administration and Scoring Manual Wechsler (2008a), appendix B tables.

2. See Appendix for a shiny app (https://wais-iv-pfit.psych.ucla.edu/) that computes $d_{\mathrm{ri}}^{2}$ . To evaluate discrepancies between the four WAIS-IV factors, one needs to compute an MD for the factor score estimates (MDFS; Yuan & Hayashi, 2010). These can also be requested from lavaan (Rosseel, 2012), which are distributed as $\chi^{2}$ with 4 *df* (see also the Appendix for a shiny app).

3. The list of possible factors that may interfere with consistent test performance is lengthy. A few examples include (a) fluctuating attention (up/down/up/down) across a 3 to 4 hr battery, such as when one test of WM is given when attention is good and another when attention is bad; (b) subclinical seizure activity affecting performance on tests in a way that is unrelated to ability in the non-ictal state; and (c) patients with either psychological disturbances that lead to idiosyncratic responses, or neuropsychological deficits in certain abilities (e.g., verbal abstraction) that may cause poor scores on some subtests (e.g., Similarities) but not other tests of the VC factor (Information, Vocabulary).
